# Supplementary material for: Unveiling the Bioactive Potential of the Invasive Jellyfish Phyllorhiza punctata Through Integrative Transcriptomic and Proteomic Analyses
Source: Biomolecules. 2025 Aug 4;15(8):1121. doi: 10.3390/biom15081121 (PMC12383608; doi:10.3390/biom15081121)
Supplement: Supplementary file 1 [file biomolecules-15-01121-s001.zip › Suplementary Material/FileS3/PEPFOLD4/XP_065069094_1227/clusters.html]

 PEP-FOLD clusters


Download archive of all models  here   

|  |  |  |
| --- | --- | --- |
| # | file | sOPEP |
| # cluster 1 - Size: 25 |  |  |
|  | XP\_065069094\_1227-model1 | -13.4981 |
|  | XP\_065069094\_1227-model1.1 | -13.4981 |
|  | XP\_065069094\_1227-model1.2 | -13.4981 |
|  | XP\_065069094\_1227-model1.3 | -13.4354 |
|  | XP\_065069094\_1227-model1.4 | -13.4354 |
|  | XP\_065069094\_1227-model1.5 | -13.4354 |
|  | XP\_065069094\_1227-model1.6 | -13.4354 |
|  | XP\_065069094\_1227-model1.7 | -13.4354 |
|  | XP\_065069094\_1227-model1.8 | -13.4354 |
|  | XP\_065069094\_1227-model1.9 | -13.4354 |
|  | XP\_065069094\_1227-model1.10 | -13.4354 |
|  | XP\_065069094\_1227-model1.11 | -13.4354 |
|  | XP\_065069094\_1227-model1.12 | -13.4354 |
|  | XP\_065069094\_1227-model1.13 | -13.4354 |
|  | XP\_065069094\_1227-model1.14 | -13.4303 |
|  | XP\_065069094\_1227-model1.15 | -13.4303 |
|  | XP\_065069094\_1227-model1.16 | -13.4303 |
|  | XP\_065069094\_1227-model1.17 | -13.4303 |
|  | XP\_065069094\_1227-model1.18 | -13.4303 |
|  | XP\_065069094\_1227-model1.19 | -13.3087 |
|  | XP\_065069094\_1227-model1.20 | -13.3087 |
|  | XP\_065069094\_1227-model1.21 | -13.3087 |
|  | XP\_065069094\_1227-model1.22 | -13.3087 |
|  | XP\_065069094\_1227-model1.23 | -13.3087 |
|  | XP\_065069094\_1227-model1.24 | -13.3087 |
| # cluster 2 - Size: 23 |  |  |
|  | XP\_065069094\_1227-model2 | -13.4914 |
|  | XP\_065069094\_1227-model2.1 | -13.4914 |
|  | XP\_065069094\_1227-model2.2 | -13.4914 |
|  | XP\_065069094\_1227-model2.3 | -13.4914 |
|  | XP\_065069094\_1227-model2.4 | -13.4914 |
|  | XP\_065069094\_1227-model2.5 | -13.4914 |
|  | XP\_065069094\_1227-model2.6 | -13.4914 |
|  | XP\_065069094\_1227-model2.7 | -13.4914 |
|  | XP\_065069094\_1227-model2.8 | -13.4914 |
|  | XP\_065069094\_1227-model2.9 | -13.4914 |
|  | XP\_065069094\_1227-model2.10 | -13.4914 |
|  | XP\_065069094\_1227-model2.11 | -13.4914 |
|  | XP\_065069094\_1227-model2.12 | -13.4914 |
|  | XP\_065069094\_1227-model2.13 | -13.4914 |
|  | XP\_065069094\_1227-model2.14 | -13.2938 |
|  | XP\_065069094\_1227-model2.15 | -13.2938 |
|  | XP\_065069094\_1227-model2.16 | -13.2938 |
|  | XP\_065069094\_1227-model2.17 | -13.2938 |
|  | XP\_065069094\_1227-model2.18 | -13.2938 |
|  | XP\_065069094\_1227-model2.19 | -13.2938 |
|  | XP\_065069094\_1227-model2.20 | -13.2858 |
|  | XP\_065069094\_1227-model2.21 | -13.2858 |
|  | XP\_065069094\_1227-model2.22 | -13.2858 |
| # cluster 3 - Size: 14 |  |  |
|  | XP\_065069094\_1227-model3 | -13.4138 |
|  | XP\_065069094\_1227-model3.1 | -13.369 |
|  | XP\_065069094\_1227-model3.2 | -13.369 |
|  | XP\_065069094\_1227-model3.3 | -13.369 |
|  | XP\_065069094\_1227-model3.4 | -13.369 |
|  | XP\_065069094\_1227-model3.5 | -13.369 |
|  | XP\_065069094\_1227-model3.6 | -13.369 |
|  | XP\_065069094\_1227-model3.7 | -13.369 |
|  | XP\_065069094\_1227-model3.8 | -13.3676 |
|  | XP\_065069094\_1227-model3.9 | -13.3676 |
|  | XP\_065069094\_1227-model3.10 | -13.3676 |
|  | XP\_065069094\_1227-model3.11 | -13.3676 |
|  | XP\_065069094\_1227-model3.12 | -13.341 |
|  | XP\_065069094\_1227-model3.13 | -13.306 |
| # cluster 4 - Size: 1 |  |  |
|  | XP\_065069094\_1227-model4 | -13.4028 |
| # cluster 5 - Size: 8 |  |  |
|  | XP\_065069094\_1227-model5 | -13.3459 |
|  | XP\_065069094\_1227-model5.1 | -13.3459 |
|  | XP\_065069094\_1227-model5.2 | -13.3459 |
|  | XP\_065069094\_1227-model5.3 | -13.3459 |
|  | XP\_065069094\_1227-model5.4 | -13.3034 |
|  | XP\_065069094\_1227-model5.5 | -13.3034 |
|  | XP\_065069094\_1227-model5.6 | -13.3034 |
|  | XP\_065069094\_1227-model5.7 | -13.2662 |
| # cluster 6 - Size: 29 |  |  |
|  | XP\_065069094\_1227-model6 | -13.3401 |
|  | XP\_065069094\_1227-model6.1 | -13.3401 |
|  | XP\_065069094\_1227-model6.2 | -13.3401 |
|  | XP\_065069094\_1227-model6.3 | -13.3401 |
|  | XP\_065069094\_1227-model6.4 | -13.3401 |
|  | XP\_065069094\_1227-model6.5 | -13.3401 |
|  | XP\_065069094\_1227-model6.6 | -13.3401 |
|  | XP\_065069094\_1227-model6.7 | -13.3401 |
|  | XP\_065069094\_1227-model6.8 | -13.3401 |
|  | XP\_065069094\_1227-model6.9 | -13.3401 |
|  | XP\_065069094\_1227-model6.10 | -13.3401 |
|  | XP\_065069094\_1227-model6.11 | -13.3401 |
|  | XP\_065069094\_1227-model6.12 | -13.3401 |
|  | XP\_065069094\_1227-model6.13 | -13.3401 |
|  | XP\_065069094\_1227-model6.14 | -13.3401 |
|  | XP\_065069094\_1227-model6.15 | -13.3401 |
|  | XP\_065069094\_1227-model6.16 | -13.3401 |
|  | XP\_065069094\_1227-model6.17 | -13.3401 |
|  | XP\_065069094\_1227-model6.18 | -13.3401 |
|  | XP\_065069094\_1227-model6.19 | -13.3401 |
|  | XP\_065069094\_1227-model6.20 | -13.3401 |
|  | XP\_065069094\_1227-model6.21 | -13.3401 |
|  | XP\_065069094\_1227-model6.22 | -13.3401 |
|  | XP\_065069094\_1227-model6.23 | -13.3401 |
|  | XP\_065069094\_1227-model6.24 | -13.3401 |
|  | XP\_065069094\_1227-model6.25 | -13.3401 |
|  | XP\_065069094\_1227-model6.26 | -13.3401 |
|  | XP\_065069094\_1227-model6.27 | -13.3401 |
|  | XP\_065069094\_1227-model6.28 | -13.3401 |
|  |  |  |
